# Biochemical and histopathological changes in Wistar rats after consumption of boiled and un-boiled water from high and low disease prevalent areas for chronic kidney disease of unknown etiology (CKDu) in north Central Province (NCP) and its comparison with low disease prevalent Colombo, Sri Lanka

**DOI:** 10.1186/s12882-020-1693-3

**Published:** 2020-01-31

**Authors:** M. G. Thammitiyagodage, N. R. de Silva, C. Rathnayake, R. Karunakaran, Kumara WGSS, M. M. Gunatillka, N. Ekanayaka, B. P. Galhena, M. I. Thabrew

**Affiliations:** 1 Medical Research Institute, Dr. Danister de Silva Mawatha, Colombo 8, Sri Lanka; 2 Department of Bio Chemistry & Clinical Chemistry, Faculty of Medicine, University of Kelaniya, Thalagolla Road, Ragama, Sri Lanka; 3 Institute of Biochemistry, Molecular Biology and Biotechnology, University of Colombo, Cumaranathunga Munidasa Mawatha, Colombo 3, Sri Lanka

**Keywords:** CKDu, NCP, BUN, Microalbumin:creatinine

## Abstract

**Background:**

Chronic Kidney Disease of unknown etiology (CKDu) is prevalent in North Central Province (NCP) of Sri Lanka. Consumption of un-boiled dug well water has been identified as one of the causative factors. This in-vivo study was performed to investigate some of the suspected factors associated with the pathogenesis of CKDu mediated via ground water.

**Method:**

Rats were given water, collected from high and low disease prevalent areas from the NCP of Sri Lanka and the results compared with those obtained from previously identified low disease prevalent area; Colombo. Blood Urea Nitrogen, creatinine, urinary microalbumin:creatinine ratio together with ALT and AST levels were analyzed and results were compared using one-way ANOVA and paired t-Test. Histopathology was analyzed using non-parametric method.

**Results:**

Rats that ingested water from New Town Medirigiriya (NTM) from high disease prevalent NCP reported significantly elevated microalbumin:creatinine ratios compared to other water sources after 8 months, whilst boiled water from NTM had been able to significantly reduce it. Histopathological findings after the 14 months experimental period revealed significantly high tubular lesion index in rats that ingested water from NCP compared to Colombo. Rats that ingested water from high disease prevalent Divuldamana (DD) from NCP showed the highest kidney lesion index though the fluoride content was relatively low in this area compared to other water sources from high disease prevalent NCP. Rats that ingested boiled and un-boiled water from NTM also developed severe lesions whilst the group from Colombo reported the lowest. Low disease prevalent area from NCP, Huruluwewa (HW) also reported elevated liver enzymes and altered renal histopathology. Association of Na^+^:Ca^2+^ ratio in the disease progression was not reflected by the current study. Compared to Colombo, high fluoride, calcium and sodium contents were observed in water from high disease prevalent areas. All the water samples were negative for heavy metals.

**Conclusions:**

Though Fluoride is a known kidney toxic agent it cannot be the sole reason for CKDu in NCP, Sri Lanka. Various toxic elements present in NCP water may contribute to different grade of kidney and liver lesions in Wistar rats.

## Background

Chronic Kidney Disease of unknown etiology (CKDu) is prevalent in Sri Lanka and is responsible for high morbidity and mortality in young adults in farming communities [1]. CKDu is diagnosed when Chronic Kidney Disease (CKD) is present without evidence of other possible causes such as diabetes, chronic or severe hypertension, snake bites, glomerulonephritis or urological diseases along with normal HbA1c levels (< 6.5%), blood pressure < 160/100 mmHg untreated, or < 140/90mm/Hg in those who are on up to two antihypertensive medications [2]. The mean age of those affected by endemic CKDu in Sri Lanka was 37.3 ± 12.5 years, and the male to female ratio was 3.3:1 [3]. It is a slow progressive, irreversible disease condition and is asymptomatic until the last stage [3]. Similar to reports from Central America, Egypt and India, CKDu is prevalent among farming communities, and is particularly seen in the Anuradhapura and Polonnaruwa districts of the North Central Province (NCP), and the Badulla district in the Uva province of Sri Lanka, with a point prevalence of 15.1–22.9% [1, 2].

Based on the results of a recent retrospective study carried out using 251 renal biopsies from patients in CKDu endemic areas of Sri Lanka, this is a tubulo-interstitial type disease condition [3]. Interstitial fibrosis, tubular atrophy with or without mononuclear cell infiltration in the interstitium have been identified as pathognomonic features of CKDu [4]. In addition, glomerular sclerosis, collapse, fibrous intimal thickening and arteriolar hyalinosis was also observed [4].

According to a study carried out in Anuradhapura, most of the population in affected areas consumes water from shallow and deep wells as the main source of drinking water. Nearly 87% of the population use either dug well or tube well water in the Anuradhapura administrative district, where most of the population in the area are affected by CKDu [5]. It has been reported that drinking water has close association with the development of CKDu among the patients reported in Anuradhapura district [6]. Ingestion of substances present in drinking water has therefore been postulated to be the cause of CKDu [7]. Although heavy metals, metalloids, pesticides and minerals have been investigated, no conclusive evidence has yet been found, regarding the responsible substances [7].

A previous study by our group revealed a significant association between consumption of dug well water collected from CKDu affected villages and the development of periglomerular and peritubular interstitial nephritis in Wistar rats, compared to rats that ingested water collected from a low disease prevalence area; Colombo, in the wet zone of Sri Lanka [8]. However, the disease prevalence is not uniform in the NCP. There is a high prevalence in some areas and a low prevalence in other areas in the same geographical region. Changes in the soil composition in these areas were suspected to be one of the causative factors for this difference [9]. High Na^+^:Ca^2+^ ratios observed in water collected from non-endemic areas such as Hurulluwewa (HW) in the NCP have been claimed to be protective in causing the disease. However, no research has been done to compare the effects of water obtained from high and low disease prevalent areas of NCP using in vivo animal models and only chemical analysis of water has been performed.

The objective of the current study is to compare the effects of un-boiled and boiled water obtained from dug wells in high and low disease prevalent areas of the NCP using Wistar rats. The effects of water obtained from Colombo was also investigated in the same model as a negative control as it has previously been established as low disease prevalent area in a different geographical location.

### Ethics clearance

Ethics clearance for this study was obtained from the Ethics Review Committee of the Medical Research Institute (MRI), Sri Lanka (No 11/2012) in compliance with the international guidelines for the use and care of laboratory animals in research.

## Methods

### Rats

*Rattus norvegicus*, Wistar strain (origin: clea-Japan) bred at the MRI under microbiologically controlled conditions were used for all the experiments. They were housed at 22 °C - 24 °C, 40–70% relative humidity and 12 h light dark cycle animal rooms and fed with a rat formula prepared based on the WHO formula prescribed by Saboudry (1980) using locally available feed ingredients [10]. Autoclaved wood shaving was used as their bedding materials and the animals were housed in a facility maintained by a barrier system with positive air pressure and 40–70% medium efficiency filters.

### Sample size calculation

Sample size per group = 2 SD^2^ (Z_α/2_ + Z_β_)^2^/d^2^ [11].

SD - Standard deviation from previous study by Thammitiyagodage et al.*,* 2017 [8]

Z_α/2_ = Z _0.05/2_ = Z 0.025 = 1.96 (From Z table) at type 1 error of 5%

Z_β_ = Z 0.20 = 0.842 (From Z table) at 80% power

d = effect size = difference between mean value

Hence,

Sample size = 2 SD^2^ (1.96 + 0.842)^2^/d^2^

= 2 (10.37)^2^ (1.96 + 0.842)^2^/15^2^

= 7.5 ~ 8

Expected attrition or death of animals: 20% from the previous study, Thammitiyagodage et al.*,* 2017 [8].

### Animal experiment

Sixty gender balanced (1:1) 12 weeks old healthy Wistar rats, males with a body weight range of 240 g ± 8 and females with a body weight range of 183.6 g ± 3.6 were selected. They were randomly divided into six groups (Group 1–6) with 10 rats per group. Groups 1–4 were given water collected from high prevalence areas in the NCP, Group 5 was given water from a low prevalence area in the NCP, whilst Group 6 was administered water from Colombo as the Control. Groups 1–3 were administered un-boiled dug well water obtained from (a) New Town Medirigiriya (NTM) in the Medirigiriya division, (b) Bisobandaragama (BB) in the Medirigiriya division, (c) Divuldamana (DD) in the Dimbulagala division respectively. Group 4 was given water from New Town Medirigiriya (NTM), collected from the same dug well as for Group 1, after boiling at 100 °C for 2 min (NTMB). Selection of dug wells for the study was based on the number of CKDu patients in the locality [8]. Water collected from the HW reservoir, from the same geographical location, but with a low disease prevalence was given to animals in Group 5. Group 6 which was considered as the negative control was given tap water collected from Colombo (CO). All the rats were housed in standard polypropylene rat cages and 3–4 rats were assigned to each experimental unit. All the rats had access to water from the respective water sources ad libitum.

### Blood collection

The rats were mildly sedated using an inhalant anaesthetic agent to immobilize them and the tail of each rat was inserted into a flask containing slightly warm water (40 °C) for one minute. They were then placed in a rat holder. A total volume of 1 mL blood was drawn from the lateral tail vein of each rat, using a 23-gauge needle and 1 mL syringe [12]. Blood samples were collected at monthly intervals and at the end of the experiment period (14 months) by a qualified veterinarian.

A 0.3mL of blood aliquot was collected into an Eppendorf tube coated with EDTA and a thin blood smear was prepared. A 0.7mL blood aliquot was separately collected into an Eppendorf tube and subsequently serum was separated by centrifuging at 12000 rpm for five minutes. Serum was stored at -20 °C until further analysis. All the experimental procedures were carried at the non-infectious animal experimental laboratory in the MRI.

### Urine collection

Rats were placed in a metabolic cage and their spot urine samples were collected into Eppendorf tubes using sterile pipettes and stored at − 40 °C until assayed.

### Sample collection for histopathology

After 14 months of the experimental period or at the end of the experimental period, all the rats were humanely euthanized using CO_2_ anaesthesia to minimize tissue changes and liver, kidney and heart samples were collected for histopathology.

### Blood and urine creatinine analysis

Commercially available creatinine Jaffe multipurpose reagents purchased from Fortress diagnostics, United Kingdom, were used for all the experiments. Serum and urine creatinine levels were estimated as per Jaffe method using previously prepared Jaffe solution (NaOH and picric acid in equal volume). Each serum samples and standards (50 μL) were gently mixed with 1000 μL of Jaffe working solution and incubated at 25 °C for 30 second. Urine creatinine was measured in the same manner by using diluted urine in distilled water at a ratio of 1:50. Quality control samples were maintained to ascertain the accuracy of the results. Absorption of the reaction mix was measured by using a Stat Fax 3300 auto biochemistry analyzer.

### Blood urea nitrogen (BUN) analysis

Commercially available Pointe scientific reagents from the United States were used and the working reagent was prepared by mixing 5 parts of reagent (R1) with 1 part of coenzyme (R2) and mixed well. Each serum sample (10 μL) was added into previously prepared working reagent (1000 μL) and the absorbance was immediately measured using a Stat Fax 3300 auto biochemistry analyzer.

### Urine Micro albumin

The urine micro albumin:creatinine ratio was estimated after 8 months of the experimental period. Human Diagnostic Reagents kits for microalbumin estimation from Germany were used for the experiment. A standard microalbumin solution at a concentration of 420 mg/L was serially diluted in 0.9% NaCl in order to obtain standard concentrations of 210 mg/L, 105 mg/L, 52.5 mg/L, and 26.7 mg/L respectively. Each diluted standards (60 μL) and urine samples (60 μL) were mixed with the buffer solution (900 μL) in individual test tubes along with a separate tube containing 0.9% NaCl (60 μL) and buffer solution (900 μL) as the reagent blank. Subsequently, each tube was added with 150 μL of antiserum, mixed and kept at room temperature for 5 min and the absorbance were measured at 340. A standard curve was plotted based on the results obtained for the dilution series of the standards and the microalbumin levels in each urine samples were calculated by using the pre-plotted standard curve.

### Serum transaminase (AST and ALT)

Commercially available Pointe scientific reagent from the United State was used to detect serum AST and ALT levels. The working reagent was prepared by mixing five parts of reagent 1 with 1 part of reagent 2 and 1000 μL of the working reagent was then added into each test tube which had been pre - warmed to room temperature. Each serum samples (100 μL) were then added and the mixture was incubated for 1 min at room temperature. AST and ALT concentrations were directly obtained by using a Stat Fax 3300 semi-automated biochemistry analyzer.

### Sample collection for histopathology

Liver, kidney and heart tissues of rats were collected into 10% formalin and subjected to histopathological investigations.

### Collection, fixation and processing of tissues for histopathology

Tissue samples were fixed for 24–48 h, and tissue sections were prepared using a rotory tissue processor (Sakura tissue-tek). The tissue samples were sequentially dehydrated through an ascending series of alcohol concentrations from 60 to 100%, by keeping them in each concentration for 2 ½ hours. Thereafter, the tissues were immersed in xylene solution for one hour and subsequently kept overnight in liquid paraffin. Finally, the tissue samples were embedded in paraffin wax solution and 4-5 μm paraffin embedded sections were prepared using a rotory microtome (Sakura ACCU- cut SRM 200). The sections were mounted on poly L-lysine coated glass slides and allowed to dry on a slide warmer for one hour.

### Haematoxylin and eosin staining procedure

The tissue sections were stained using the method described by Alan & Ian in 1996 [13]. The 5 μm thick sections that were embedded and mounted previously were deparaffinized in xylene and rehydrated in descending concentrations of alcohol in a sequential manner. These were immersed in absolute alcohol, 90% alcohol, 80% alcohol, 70% alcohol and finally in distilled water. Samples were kept for 2–3 min in each solution. Thereafter, the sections were stained in Harris’s Haematoxylin for 15 min and washed in running tap water until the brown colour disappeared. The slides were then immersed in a lithium carbonate solution for 10 s and washed in tap water. Washed slides were stained with Eosin (30 s) with agitation followed by washing with tap water. Sections were dehydrated in 70%, 90% and two changes of absolute alcohol with agitation (one minute each). Finally, the sections were cleared in xylene, and mounted and examined under a light microscope [13].

### Histopathological grading the renal lesions

In order to assess the significance levels of tubulo-interstitial (TI) lesions, the following procedure was adopted. Using previously stained slides, mild, moderate and severe tubular lesions were calculated in ten randomly selected fields under the (10 × 40) magnification. The TI lesion score was assessed and graded according to the scale introduced by Toblli et al (Grade 0 = 0%, 1 + = 1–25%, 2 + =26–50%, 3 + = 51–75% and 4 + = 76–100%) [14].

### Analysis of water samples

Three water samples collected from each well and the water samples collected from HW and the tap water samples collected from Colombo were assayed using standard methods described by the American Public Health Association (APHA) [15]. The fluoride level was assayed using the APHA 4500-PC method, the total iron by the APHA 3111B or the direct air -acetylene flame method, the calcium levels by the APHA 3500 Ca -D method, the sodium by the APHA 311B method, the cadmium levels by the standard 3113B method and the arsenic levels using the APHA 3114 C method [15].

### Statistical analysis

Statistical analysis was performed using the SPSS statistical package. Baseline parameters of bio-chemical data were compared with the values obtained after 14 months of experimental period using paired t-Test. The comparison of the data obtained from different test groups and control groups after 14 months of experimental period were performed using one-way ANOVA with post hock. Nonparametric values of histopathological data were converted to parametric values and tested using the Wilcoxan sign rank test.

## Results

### Animal experiment

Gender variation was not considered as no differences were observed in bio chemical parameters and histopathology. The baseline values of serum creatinine, BUN, microalbumin: creatinine, ALT and AST levels are summarized in Table 1.

**Table 1:** Biochemical parameters at the baseline. Values are expressed as a mean ± SEM (*n* = 30)

Biochemical parameter	Unit	Value	
Serum creatinine	[mg/dL]	0.54 ± 0.02	
BUN	[mg/dL]	39.02 ± 1.74	
Microalbumin: creatinine	[mg/g]	32.32 ± 5.9	
AST	[U/L]	87.33 ± 20.06	
ALT	[U/L]	33.51 ± 2.83	

Microalbumin:creatinine ratios were evaluated after 8 months and the other parameters were re-evaluated after 14 months and the results are summarized in Table 2.

**Table 2:** Microalbumin:creatinine ratios and other biochemical parameters after 8 and 14 months of experimental period

Bio Chemical Parameter	Unit	NTM	NTMB	DD	BB	HW	CO
Serum creatinine	[mg/dL]	0.80 ± 0.03	0.80 ± 0.04	0.85 ± 0.03	0.86 ± 0.02	0.79 ± 0.04	0.76 ± 0.04
BUN	[mg/dL]	36.37 ± 1.36	36.37 ± 1.36	39.42 ± 2.74	36.54 ± 3.53	32.88 ± 1.68	35.27 ± 0.896
Microalbumin:creatinine	[mg/g]	140.28 ± 28.29	59.35 ± 5.05	67.39 ± 8.09	30.23 ± 9.48	70.03 ± 11.62	19.08 ± 7. 14
AST	[U/L]	97.8 ± 7.5	92.3 ± 6.0	93.6 ± 16.1	101.0 ± 5.5	175.8 ± 23.1	88.41 ± 16.37
ALT	[U/L]	39 ± 4.5	42 ± 4.5	32.2 ± 8.1	40.6 ± 2.5	123.4 ± 30.5	40.88 ± 18.33
Histology (non-affected / affected)	%	0.46 ± 0.18	0.28 ± 0.07	0.48 ± 0.14	0.31 ± 0.07	0.39 ± 0.14	1.0

*NTM* New Town Medirigiriya, *NTMB* New Town Medirigiriya Boiled, *DD* Divuldamana, *BB* Bisobandaragama, *HW* Huruluwewa, *CO* Colombo. Values are expressed as a mean ± SEM (*n* = 60).

When compared to the base line creatinine values, all the rats in different groups reported statistically significant high serum creatinine levels (*p* < 0.05) at the end of 14 months. No significant differences were observed between groups after 14 months (*p* > 0.05). A comparison of the baseline BUN levels with values obtained after 14 months showed that there were no significant differences (p > 0.05). Comparison of the groups after the 14-months experimental period indicated that rats that ingested water from DD and HW showed slightly elevated BUN levels compared to other groups, which was not significant. The lowest BUN levels were observed in rats that ingested water from CO. Results are summarized in (Table 2). Urine microalbumin:creatine ratios after 8 months were compared with their base line parameters using the paired t-Test. With the exception of rats that ingested water from BB and CO, the other groups (NTM, NTMB, DD and HW) had significantly high urine microalbumin:creatinine compared to their baseline levels. (Table 2). The experimental groups NTMB, BB, DD, HW, CO had statistically significant low urine microalbumin:creatinine ratios compared to rats that ingested un-boiled water from NTM (*P* < 0.05). Rats that ingested boiled water from NTM had statistically significant low urine microalbumin:creatinine ratios compared to rats that ingested un-boiled water from NTM (*p* = 0.007). Rats from DD and HW had significantly high urine microalbumin:creatinine compared to Colombo. Colombo reported the lowest ratio.

Base line levels of AST and ALT of three-months-old Wistar rats are summarized in Table 1. When compared with base line values, no significant increase of ALT and AST levels were observed in any group except in the HW group (Table 2). Rats that ingested water from HW had significantly high ALT and AST levels compared to other experimental groups after 14 months and significance was (*p* = 0.054) and *p* = (0.02) respectively.

### Histopathological changes in kidney tissues

**Fig. 1:**
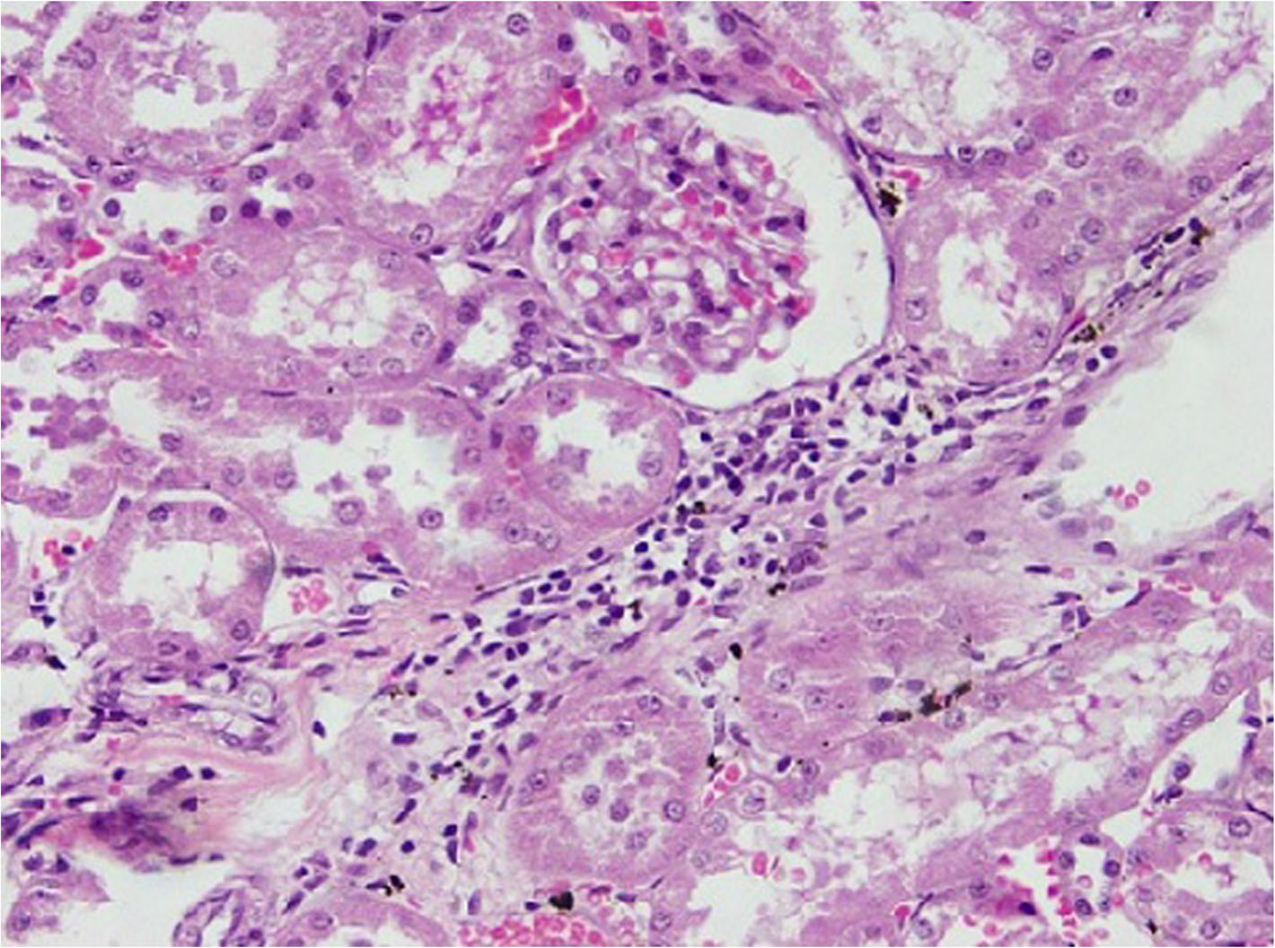
Chronic inflammatory cell infiltrates around the kidney tubules with tubular degeneration. Kidney tubules of rat tissue under (H&E) stain with (10 × 40) magnification

**Fig. 2:**
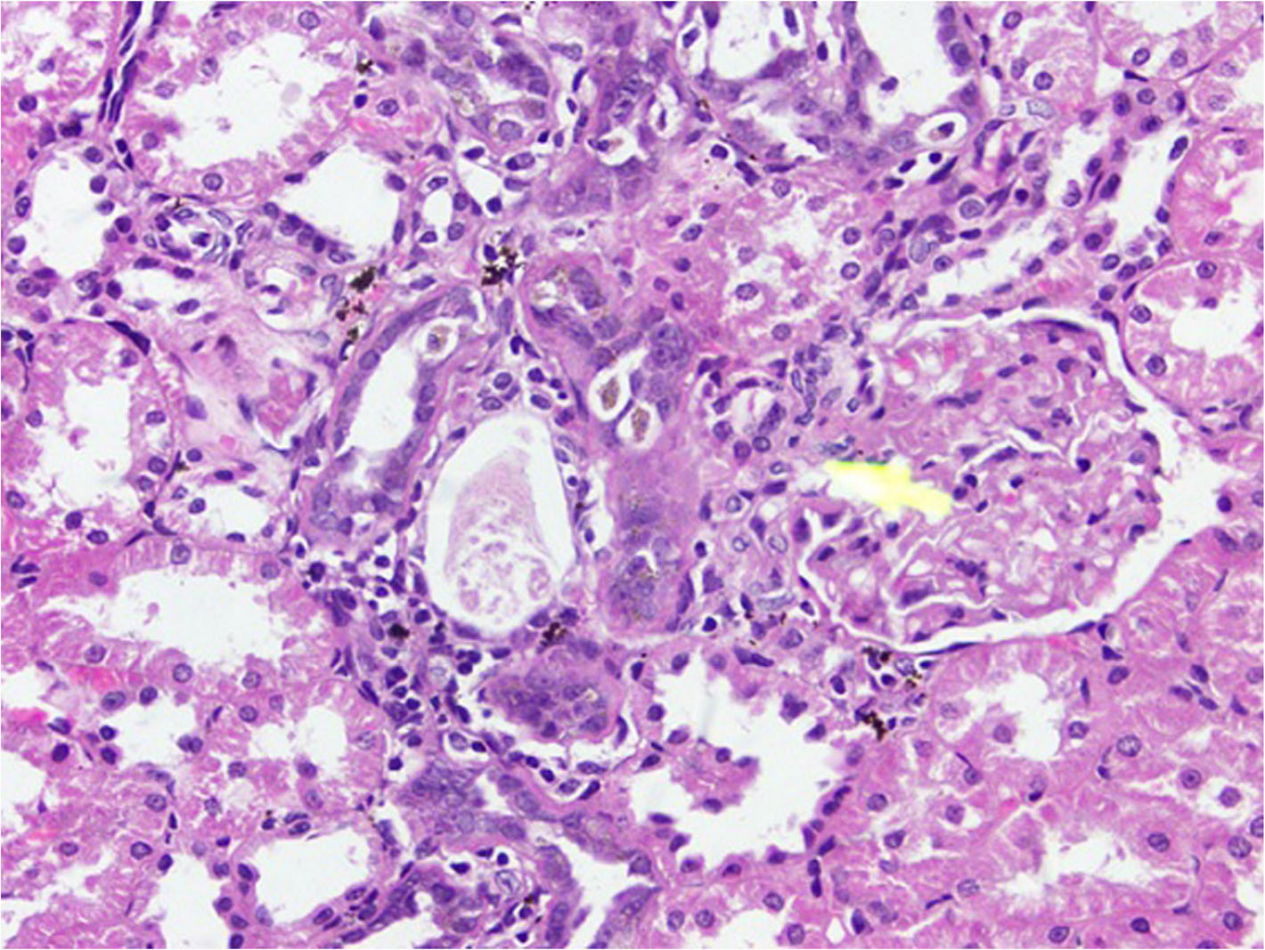
Chronic inflammatory cell infiltrates around the kidney tubules with tubular degeneration and regeneration. Degenerated kidney tubules of rat tissue under (H&E) stain with (10 × 40) magnification

**Fig. 3:**
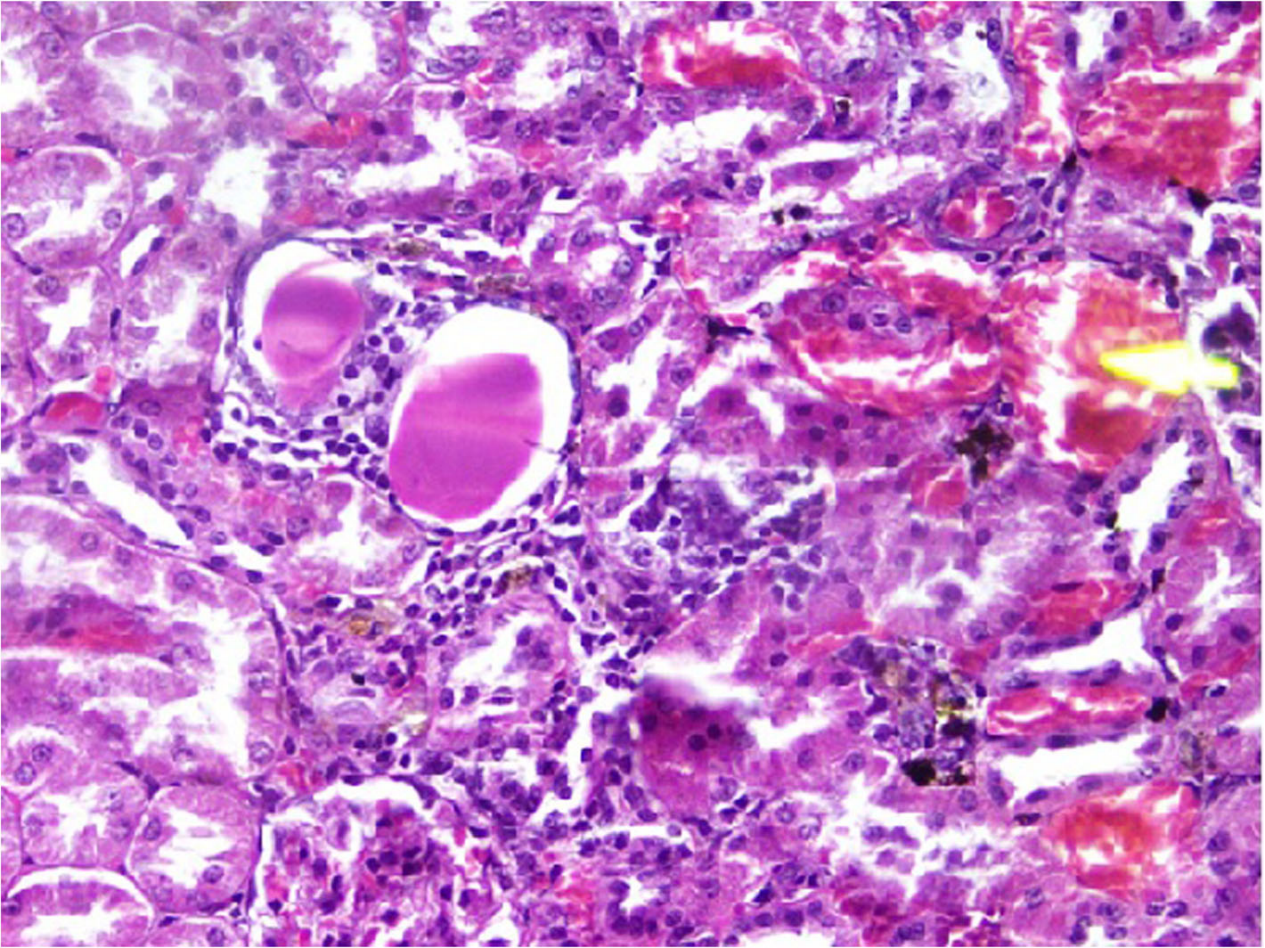
Degenerated kidney tubules with chronic inflammatory cell infiltrates. Peritubular chronic cell infiltrations with prominent tubular destruction under (H&E) stain with (10 × 40) magnification

**Fig. 4:**
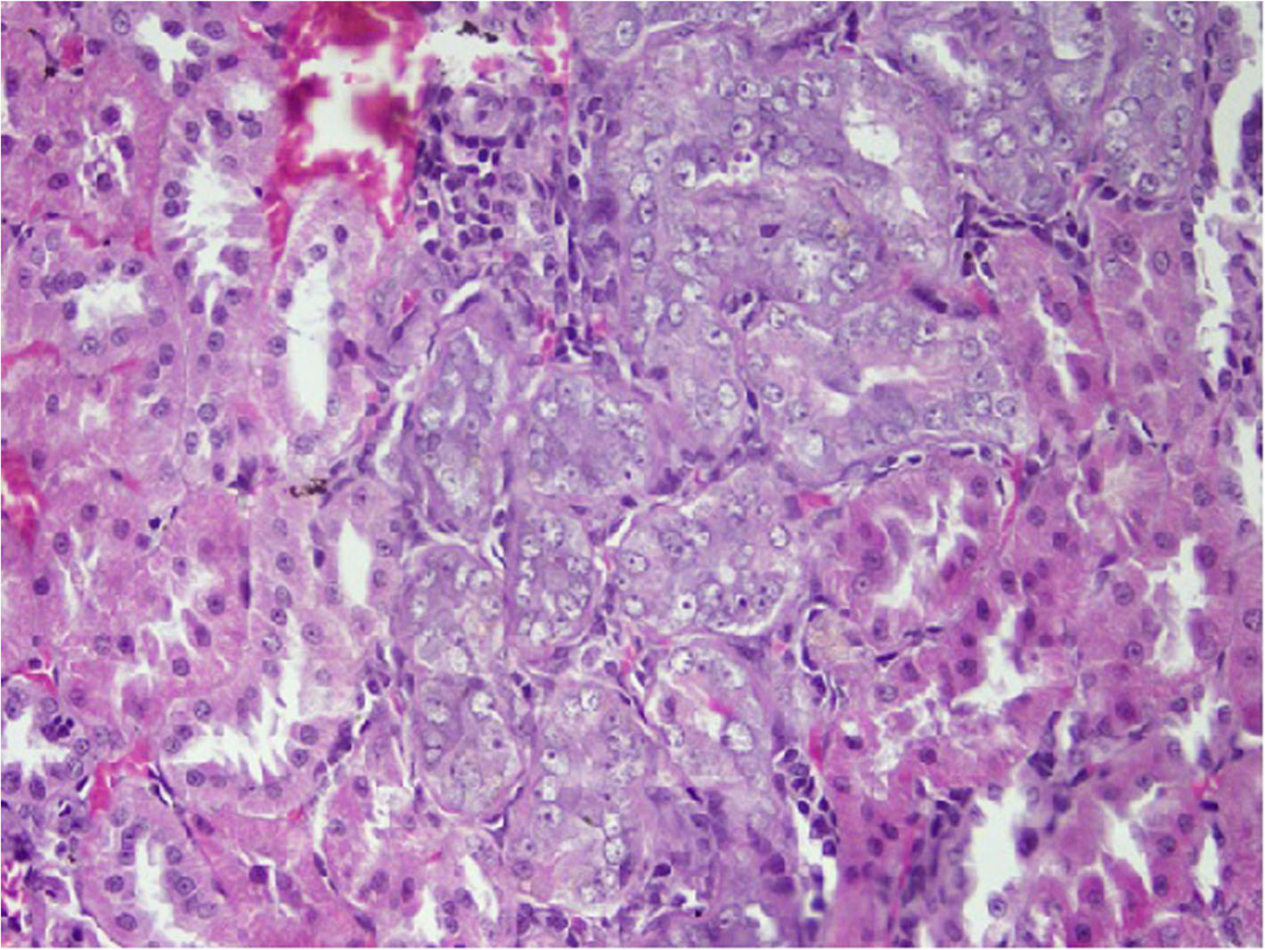
Areas with tubular regeneration. Rat kidney tubules with tubular regeneration under H&E stain (10 × 40)

Rats from NTM had chronic, moderate to severe interstitial inflammatory foci. In a few rats, tubular degeneration, tubular regeneration and fibrosis were also observed (Figs. 1, 2, 3, 4). Rats from NTMB also developed mild to severe chronic interstitial inflammatory foci and in some rats, tubular degeneration was also observed (Fig. 1).

In rats from DD in addition to inflammatory foci, very prominent tissue destruction and fibrosis were observed (Fig. 3). Rats from BB also developed mild to moderate peritubular chronic inflammatory foci whilst rats that ingested water from HW had mild peritubular chronic interstitial inflammatory foci. Of the rats that ingested water from CO, a few developed very mild inflammatory foci. Data of the tubular lesion index was analyzed by a non - parametric method using two independent sample t- Test. All the rats that ingested boiled and un-boiled water from high disease prevalent areas had a statistically significant tubular lesion index compared to rats from Colombo (*p* < 0.05). No statistically significant difference was observed in rats that ingested boiled and un-boiled water from NTM (*p* > 0.05) at tissue level. In order to understand the distribution of the lesions across the kidney tissues, 10 non overlapping microscpic fields were observed under the 10 × 40 magnification and the non affected fields to affected field ratios were calculated. According to the results, a majority of rats that ingested water from the CO group had more unaffected areas compared to affected regions (Table 2). Rats from NTM had significantly more non affected fields than DD. In NTM, focal chronic peritubular lesions with tubular destruction and regenerations were more prominent than mild scattered peritubular lesions observed in HW. In contrast DD had very severe chronic peritubular lesions which were equally distributed troughout the entire kidney. In CO most of the kidney tissue remained unaffected whilst very mild scattered lesions were observed in only one field.

### Liver lesion

**Fig. 5:**
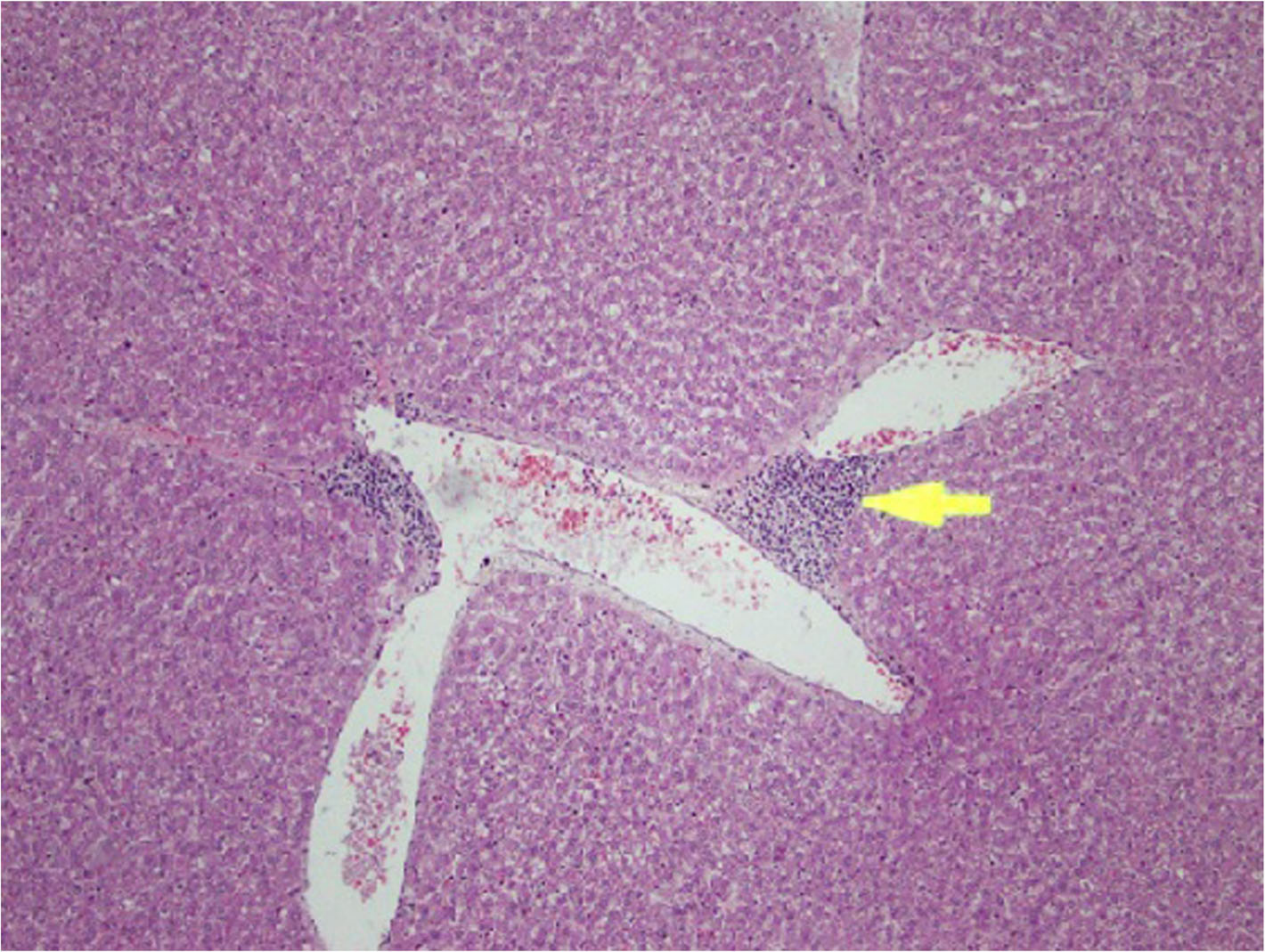
Chronic Inflammatory cell infiltrates around the portal tracts in liver sections. Chronic inflammatory cell infiltrates around the portal tracts under (H&E) stain and (10 × 4)

**Fig. 6:**
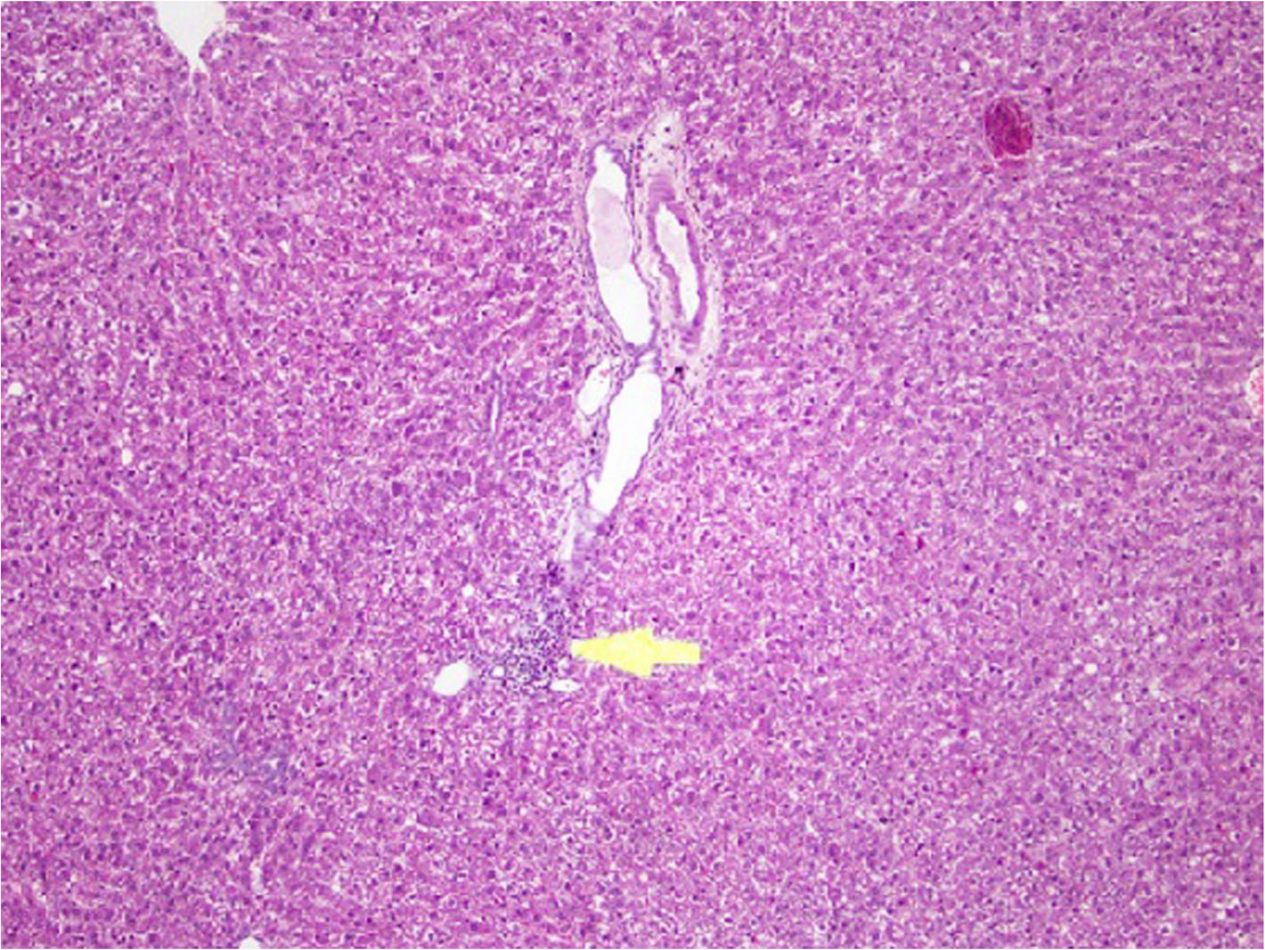
Inflammatory cell foci in liver parenchyma. Mild chronic inflammatory foci in the liver parenchyma under H&E stain and (10 × 4) magnification

In some rats from HW, moderate inflammatory foci were observed, whilst in a few rats, mild to moderate inflammatory lesions were observed in the portal tracts. In one rat chronic active hepatitis including inflammatory cells in the liver parenchyma was observed confirming the statistically significant increase in liver enzyme levels. In most rats from NTM, mild focal peri portal inflammatory cell infiltrates were observed whilst in rats from NTMB, mild to severe chronic inflammation in the portal tracts were observed (Fig. 5). In most rats from BB, mild focal chronic inflammatory cell infiltrates were observed. All the rats from DD had mild focal inflammatory foci in the portal tract or parenchyma; some lesions were found to be chronic. In rats from Colombo, normal hepatic architecture was preserved in a majority whilst in only a few animals very localized mild to severe chronic inflammatory foci were observed in the liver parenchyma but not in the portal tracts (Fig. 6).

#### Heart

In all the rats normal tissue architecture was preserved.

### Water analysis

High fluoride and calcium contents were observed in water collected from NTM. Boiling of water did not produce any significant changes in the composition (NTMB). Lowest fluoride, calcium and sodium contents were observed in water collected from CO. Results of water analysis are summarized in Table 3.

**Table 3:** Water analysis from high and low disease prevalent areas for CKDu in NCP and from Colombo

	NTM	NTM boiled	BB	DD	HW	Colombo
Fluoride (as F)	1.3 mg/dL	1 .3/mg/dL	0.56 mg/dL	0.34/mg/dL	0.30 mg/dL	0.093 mg/dL
Total Iron (as fe) (mg/L)	ND	ND	ND	ND	ND	ND
Calcium (as Ca)	10.8 mg/L	12.5 mg/L	9.8 mg/L	8.4 mg/L	6.2 mg/L	4.7 mg/L
Na+/Ca2+	5.09	4.56	2.65	2.14	4.19	0.55
Sodium (as Na)	55	57	26	18	26	2.6
Arsenic (as as) **(**0.001 mg/L)	ND	ND	ND	ND	ND	ND
Cadmium (as Cd) (0.001 mg/L)	ND	ND	ND	ND	ND	ND

NTM: NTMB boiled, BB, DD, HW, CO water samples analysis at the Industrial Technology Institute, Colombo, Sri Lanka

Not detectable levels: Total Iron; below 0.1, Arsenic: below 0.001, Cadmium: below 0.001

New Town Medirigiriya (NTM), Boiled water from Medirigiriya (NTMB), Bisobandaragama (BB), Divuldamana (DD) from high disease endemic areas for CKDu of unknown origin and Huruluwewa (HW) and Colombo (CO) that were used as controls from low endemic areas (*n* = 10)

All the values are expressed with Standard Error (SE) and precisions were done in 95% confidence intervals (*p* < 0.05)

## Discussion

The demography of the current study was based on previous epidemiological findings and a questionnaire-based study performed by the investigators [16]. Majority of the people in the selected villages consume dug well water (83%) whilst 8% consume water from tube wells, 7% from pipe borne water, 1% from natural water tanks distributed in the area and 1% from all the above sources. Of these, 81% consume un-boiled water and 19% consume boiled water irrespective of its origin. As the majority consume un-boiled dug well water (83%), the authors hypothesized that including a boiled water sample from an area with the highest disease burden (NTM) will have some impact on the experimental design. As such, dug well from NTM was selected to represent boiled as well as un-boiled water considering the very high disease burden in the locality [8, 17].

There was a significant increase in serum creatinine levels in all the rats compared to their baseline values (Table 1 and Table 2). Usually, rat serum creatinine levels are in the range of 0.4–0.8 mg/dL [18]. However, no significant differences were observed in BUN of any groups compared to their baseline values. Usually rat BUN is resistant to age and gender related variation and it is within the range of 15–22 mg/dL [18, 19]. Low serum creatinine and BUN levels were expected from CO as the incidence of CKDu in the province is low but this was not observed in our study [20]. However, further evidence for kidney dysfunction can be assessed by the urine microalbumin:creatinine ratio. When compared to their baseline values, rats that ingested water from NTM, NTMB, DD and HW had significantly high urine microalbumin:creatinine ratios. When comparisons were done between different groups after 8 months, the highest microalbumin:creatinine ratios were observed in rats that ingested unboiled water from NTM. This can be correlated to various toxic compounds dissolved in un-boiled dug well water. The urine microalbumin:creatinine ratios in rats that ingested boiled water from NTM was significantly lower than rats that ingested un-boiled water from NTM. In addition to measured mineral constituents, there may be some other temperature sensitive toxic components in the un-boiled dug well water in the NCP contributing to the altered kidney functions in rats. Values obtained from these experiments were also compared with the microalbumin:creatinine ratios of rats that had been used for other interventions in order to find out the ratios observed in Wistar rats in terms of grading the severity. In a study carried out using 28-week-old Wistar rats in an obesity trial, the control group of rats showed a microalbumin:creatinine ratio of 19.62 ± 7.81 μg/mg whist in an obese group it was 54.47 ± 23.2 μg/mg [21]. Accordingly, most of the rats in our experimental groups have mild to moderate microalbumin:creatinine ratios except for rats that ingested water from NTM. Microalbuminuria is an early marker of renal damage which progresses into proteinuria which is a major complication in advanced kidney disease. Overall, the data provides evidence for the presence of altered kidney functions in rats maintained on water from the NCP.

As expected, a significantly high number of kidney lesions were observed in all the rat groups that ingested boiled as well as un-boiled water from high disease prevalent areas. Kidney lesions were also observed in rats that ingested water from the low disease prevalent HW but the severity of the lesions was less compared to rats that consumed water from high disease prevalent areas. Various unknown toxic elements dissolved in water from the NCP may contribute to the disease burden of CKDu in the NCP, Sri Lanka similar to the in vivo Wistar rat model.

In the previous animal experiment carried out using water from the same wells in CKDu affected areas, in addition to peritubular lesions, periglomerular lesions were also observed together with chronic active hepatitis and hepatocellular carcinoma [8]. The duration of that experiment was 15 months and rats that ingested water from high disease prevalent areas started dying. As such, this experiment was terminated after 14 months. This may be a reason for not detecting very severe lesions in the present study. Another major observation in the previous animal experiment using water collected from the same wells in the NCP (NTM, BB, DD) were much higher calcium levels (e.g. 46 mg/L, 40 mg/L and 21 mg/L) whilst having almost the same fluoride levels (e.g. 1.3 mg/dL, 0.57mg/dL and 0.48mg/dL) [8]. But in the current study we observed much lower level of calcium concentrations in the selected wells and that may be correlated to seasonal variations observed in the country due to the monsoon rain, temperature and pH gradients (Table 3). This kind of seasonal variations can have significant impact on other un measured toxic elements present in water bodies in the NCP and can contribute in varying degrees to the CKDu burden as observed in the current in-vivo animal model.

A study was carried out by Perera et al on chemical speciation of water from CKDu affected and non-affected areas in Sri Lanka with the main focus on the chemical speciation of drinking water which is important in order to understand the bio availability, chemical toxicity and environmental impact of the metal ions, Pb^2+^, Al ^3+^, Cu^2+^, Cr^3+^ and Cd^2+^ showed the highest variation with pH and temperature. It was noted that formation of different species is high with pH changes, and the formation of different species with F^−^ ions in affected areas may have a significant impact on CKDu in endemic areas [22]. In another Sri Lankan study carried out by Wasana et al using 120 numbers of Institute of Cancer Research (ICR) mice, the synergistic effect (s) of heavy metals, aluminum, fluoride, arsenic and hardness in water on kidney tissues was found to be more significant rather than individual elements existing in the water [23]. High Na^+^:Ca^2+^observed in non-endemic areas such as HW is claimed to be protective due to low disease incidence rates in such areas [24]. High Na^+^:Ca^2+^ ratio was found in both low disease prevalent HW as well as in high disease prevalent NTM in current study (Table 3). A study conducted in Polonnaruwa district which analyzed ground water samples collected to represent CKDu positive and CKDu negative people in the area revealed, much lower Na^+^:Ca^2+^ ratios than the values reported by Chandrajith et al. The mean Na^+^:Ca^2+^ ratios reported from ground water samples from where CKDu patients were reported from Polonnaruwa was 0.66 whilst in areas where CKDu patients were not reporting was 0.57 respectively [25]. Whatever the causative reason, a low tubular lesion index was observed in rats that ingested un-boiled water from HW compared to rats that ingested boiled and un-boiled dug well water from high disease prevalent areas of the NCP. The highest number of chronic lesions were observed in rats that ingested water from the high disease prevalent DD but rats that ingested boiled and un-boiled water from NTM also reported severe lesions. Rats that ingested boiled water from NTM was compared with the rats that ingested water from CO and there was no significant difference observed in the non-affected: affected kidney tissue ratios. However, rats that ingested un boiled water from BB also showed no significant difference compared to rats that ingested water from CO, which had the lowest lesion index. However, though fluoride levels were much lower in NTM, the most severe lesion index was observed in rats that ingested water from DD. Though fluoride is a known nephro toxic agent, fluoride cannot be the sole reason for CKDu in NCP, Sri Lanka.

In rats that ingested water from HW, a significant rise in ALT and AST levels was observed compared to their baseline values (*p* = 0.000) after the 14 months although no such increases were observed in the other groups. (Table 2). Rat serum ALT and AST levels are usually stable and do not vary with age and gender and are usually higher than the normal range in humans when the same detection methods are employed [18]. Though serum ALT and AST levels were significantly elevated in rats that ingested water from HW, it was within the normal range.

Significantly altered liver architecture was also observed in the rats that ingested water from HW. Rise in ALT and especially AST indicates chronic hepatocellular dysfunction. These findings together with the histological data reveal the presence of prominent liver damage in the HW group which is not found in any other group. This suggests that water from HW contains substance(s) which, with prolonged exposure, leads to hepatocellular dysfunction. Rats that ingested water from DD also had very severe liver lesions compared to the other groups from high disease prevalent areas.

Aflatoxin is a potential hepatotoxic agent found in animal feeds specially in finished commercial animal feeds and feed ingredients in developing countries. However, the rats in the current study were maintained on a ration prepared using locally purchased feed ingredients. According to a study carried out in Guyana, out of 30 samples of finished feed samples 5 samples had aflatoxins whilst in feed ingredients, out of 30 samples only three samples were positive [26]. The contamination of animal feeds and feed ingredients by aflatoxin is very low, and its impact on the liver should have been equally distributed among all the experimental groups in our study as all groups consumed the same food. There is a possibility of having mycotoxins in surface water bodies such as HW and dug well water in selected wells. Many cyanobacterial blooms are highly toxic and prolonged exposure can cause liver as well as kidney damage. Common toxic agents secreted by cyanobacteria were identified as peptide hepatotoxins. These peptide hepatotoxins are secreted by many forms of locally present cyanobacteria [27]. Hepatotoxins are not equally distributed among different water bodies and can contribute to different grades of liver and kidney lesions in experimental groups.

However, there are limitations in animal models due to differences in the liver enzyme system in human and rats as certain enzymes are deficient in rat liver [28]. That may be considered as the reason for not detecting liver lesions in CKDu patients in NCP, Sri Lanka.

## Conclusion

Various toxic elements dissolved in NCP water may contribute to different grades of kidney and liver lesions in Wistar rats. Boiling of water and Na^+^:Ca^2+^ has no significant impact on disease pathogenesis. Though fluoride is a known nephrotoxin, it cannot be the sole reason for CKDu in the NCP, Sri Lanka. According to results generated in the current study it is highly suggestive that CKDu in NCP is multifactorial and a multisector approach is recommended in order to reduce the incidence rate of CKDu.

## Data Availability

Data generated or analyzed during this study are included in this manuscript [Table 1, Table 2, Table 3.].

## Abbreviations

ALT: Alanine Amino Transferase
APHA: American Public Health Association
ANOVA: Analysis of Variance
AST: Aspartate Amino Transferase
BB: Bisobadaragama
BUN: Blood Urea Nitrogen
CKDu: Chronic Kidney Disease of unknown etiology
CO: Colombo
DD: Divuldamana
EDTA: Ethylene Diamine Tetra Acetic acid
HW: Huruluwewa
ICR: Institute of Cancer Research
MRI: Medical Research Institute
NTM: New Town Medirigiriya
NTMB: New Town Medirigiriya Boiled
NCP: North Central Province
TI: Tubular Interstitial
WHO: World Health Organization
